# Finite element analysis of two cephalomedullary nails in treatment of elderly reverse obliquity intertrochanteric fractures: zimmer natural nail and proximal femoral nail antirotation-ΙΙ

**DOI:** 10.1186/s13018-019-1468-3

**Published:** 2019-12-10

**Authors:** Jian Chen, Jian-xiong Ma, Ying Wang, Hao-hao Bai, Lei Sun, Yan Wang, Bin Lu, Ben-chao Dong, Ai-xian Tian, Xin-long Ma

**Affiliations:** 1 Department of Orthopedics, Tianjin Medical University General Hospital, Tianjin, 300052 People’s Republic of China; 2 Institute of Orthopedics, Tianjin Hospital, Tianjin University, Tianjin, 300050 People’s Republic of China

**Keywords:** Finite element analysis (FEA), Reverse obliquity intertrochanteric fracture, Fracture fixation, Zimmer natural nail (ZNN), Proximal femoral nail antirotation-II (PFNA-II)

## Abstract

**Background:**

More elderly patients are suffering from intertrochanteric fractures. However, the choice of internal fixation is still controversial, especially in the treatment of unstable intertrochanteric fracture; thus, previous implants continue to be improved, and new ones are being developed. The purpose of our study was to compare the biomechanical advantages between the zimmer natural nail (ZNN) and proximal femoral nail antirotation-II (PFNA-II) in the treatment of elderly reverse obliquity intertrochanteric fractures.

**Methods:**

A three-dimensional finite element was applied for reverse obliquity intertrochanteric fracture models (AO31-A3.1) fixed with the ZNN or PFNA-II. The distribution, peak value and position of the von Mises stress and the displacement were the criteria for comparison between the two groups.

**Results:**

The stresses of the internal fixation and femur in the ZNN model were smaller than those in the PFNA-II model, and the peak values of the two groups were 364.8 MPa and 171.8 MPa (ZNN) and 832.3 MPa and 1795.0 MPa (PFNA-II). The maximum amount of displacement of the two groups was similar, and their locations were the same, i.e., in the femoral head vertex (3.768 mm in the ZNN model and 3.713 mm in the PFNA-II model).

**Conclusions:**

The displacement in the two models was similar, but the stresses in the implant and bone were reduced with the ZNN. Therefore, the ZNN implant may provide biomechanical advantages over PFNA-II in reverse obliquity intertrochanteric fractures, as shown through the finite element analysis. These findings from our study may provide a reference for the perioperative selection of internal fixations.

## Introduction

With the increase in the elderly population, the incidence of intertrochanteric fractures caused by low-energy trauma is increasing as well [1]. How to obtain good reduction and fixation of fracture and promote patients’ early activities has always been the aim of orthopaedic surgeons. Therefore, we should focus on the stability and intensity of internal fixation for treatment [2].

PFNA has been reported to have good clinical outcomes [3, 4]. However, during treatment of some patients with large anterior bow radius of the femur, as the short PFNA is a straight nail, problems such as mismatch of anatomical configuration with the proximal femur, postoperative pain of the proximal thigh and stress fracture of the distal end of the main nail will occur [5, 6]. PFNA-II (Depuy Synth, USA) was produced based on some improvements to the standard PFNA. For example, the diameter of the proximal end of the main nail is reduced from 17 to 16.5 mm, the diameter of the helical blade is reduced from 10.55 to 10.3 mm and the valgus angle of the main nail is reduced from 6 to 5°. After conducting a multi-centre prospective study, Sawaguchi et al. [7] concluded that PFNA-II is safe and effective in patients with unstable femoral intertrochanteric fractures, but the short PFNA-II is also a straight nail and still does not match the femur to some extent. To solve this problem, a new implant called the ZNN was developed (Zimmer, Germany). This new type of implant accommodated the entire anatomical structure of the proximal femur. The short nail has a radius of curvature of 1275 mm and 15° anteversion with different centre-column-diaphyseal (CCD) angles (125°/130°). The lag screw diameter is 10.5 mm. It also has been proven that ZNN has good clinical results [8].

There have been many biomechanical studies on the fixation of intertrochanteric fractures [9, 10], but few studies have compared the biomechanical performance between the ZNN and PFNA-II. As far as internal fixation is concerned, it is not clear which device has better mechanical stability. Reverse obliquity intertrochanteric fracture is an unstable intertrochanteric fracture with a high incidence of complications and failure rate. Therefore, the aim of our study was to use FEA to compare the biomechanical performance between ZNN and PFNA-II in the treatment of reverse obliquity intertrochanteric fracture (AO/OTA classification 31-A3.1) and to provide a reference for the perioperative selection of internal fixations.

## Methods

### Three-dimensional modelling of the femur and implant

**Fig. 1:**
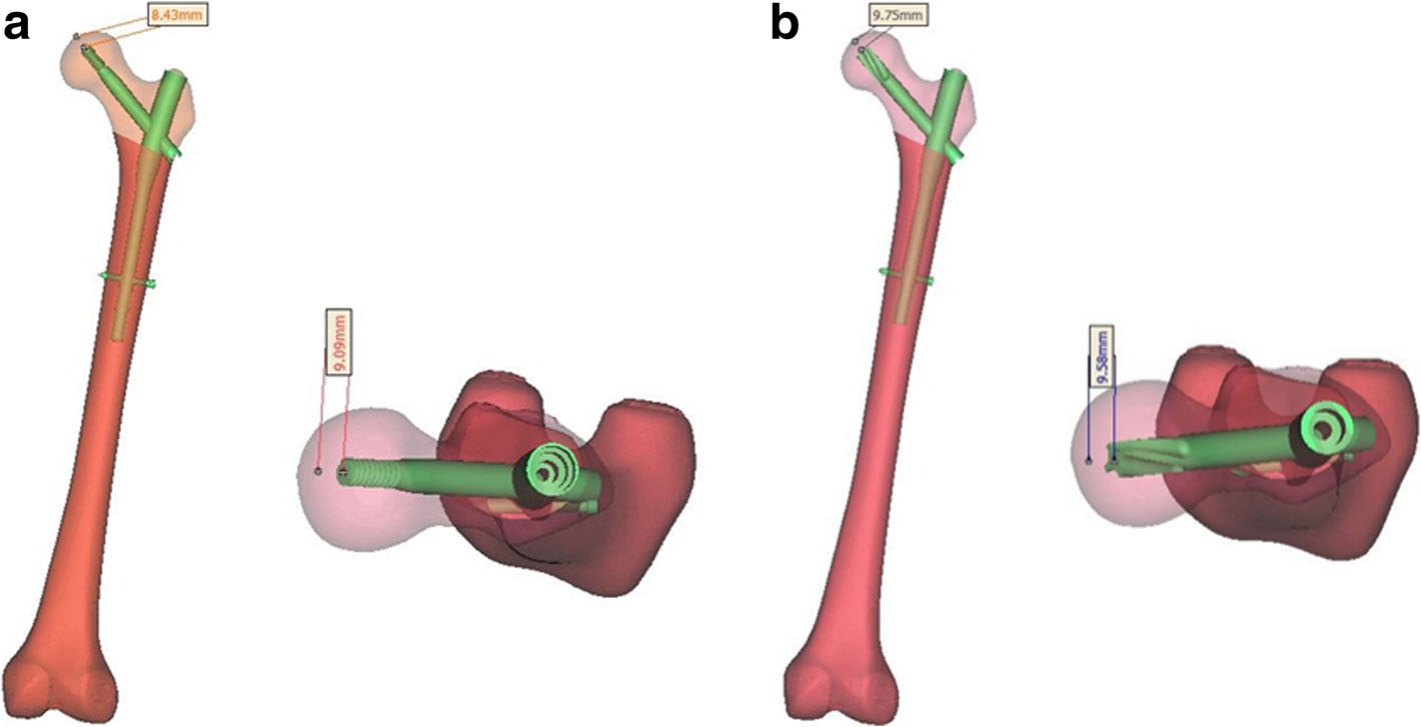
Establishment of geometric model of reverse obliquity intertrochanter fracture (AO/OTA type 31-A3.1) fixed by ZNN and PFNA-II, and measured the TAD (**a** 17.52 mm in ZNN group, **b** 19.33 mm in PFNA-II group)

One healthy Chinese male volunteer was chosen: age 67 years, weight 70 kg, height 169 cm. The X-ray appearance of the femur was normal, with no signs of femoral diseases or deformities. The femur was scanned with a 64-slice spiral CT (GE, USA), and the data were saved in DICOM format. Then, Mimics 17.0 software (Materialise, Belgium) was used to reconstruct three-dimensional (3D) models of the femur from the CT images. Using virtual osteotomy, we established a model of unstable intertrochanteric fracture corresponding to the Muller AO classification 31-A3.1 [11]. The geometrical dimensions of the ZNN (length 180 mm, diameter 10 mm, 4° proximal lateralization angle, 15° anteversion, 1275-mm anterior bow radius, lag screw length 95 mm, CCD angle 130°) and PFNA-II (length 170 mm, diameter 11 mm, 5° proximal lateralization angle, helical blade length 95 mm, CCD angle 130°) were obtained from the implant manufacturer’s catalogue. The dimensions were then input into a computer-aided design (CAD) program, SolidWorks 2012 (Dassault, France), for the reconstruction of 3D models. Later, a geometric model of the implants was assembled with a 31-A3.1 fracture model, and the tip-apex distance (TAD) was controlled within 20 mm (Fig. 1).

The geometric fracture model of the femur and internal fixation was imported to the finite element analysis pre-processing software Hypermesh 13.0 (Altair, USA) to draw the mesh. The model was meshed with 4-node tetrahedron elements. Convergence tests were performed to determine the optimum maximum element size. After the convergence measurement, the mesh size was determined to be 1 mm.

**Fig. 2:**
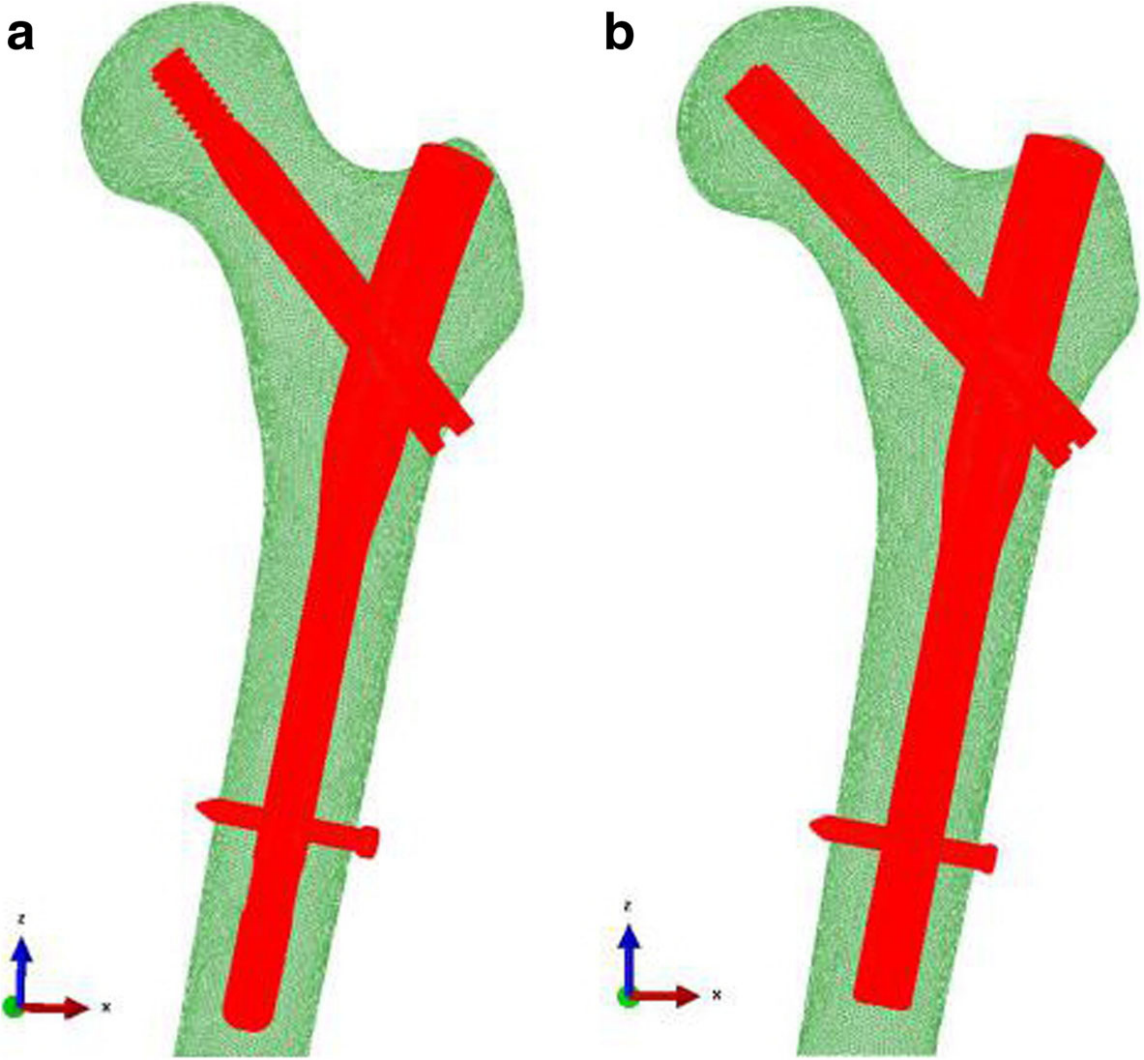
Three-dimensional finite element model (**a** ZNN model, **b** PFNA-II model)

Thereafter, the 3D finite element fracture models based on the ZNN and PFNA-II were established (Fig. 2). The number of nodes and elements of the two models are shown in Table 1. After the complete construction of the 3D computer models, calculations were performed with the FE analysis software Abaqus 6.14 (Dassault, USA).

**Table 1:** Number of nodes and elements in the meshes of the two groups of models

	ZNN	PFNA-II
Number of nodes	222463	221253
Number of elements	1059288	1051216

### Material properties

All materials were assumed to be linearly elastic, isotropic and homogeneous [12]. Two implants, both made of titanium alloy, were used. Table 2 shows the material properties of the femur and implant materials [13, 14].

**Table 2:** Material properties used in the simulations in this study

Material	Young’s modulus (Mpa)	Poisson’s ratio
Cortical bone	17,000	0.33
Cancellous bone	1000	0.3
ZNN (Ti-6Al-4 V)	114,000	0.34
PFNA-II (Ti-6Al-7NB)	110,000	0.35

### Model validation

To validate the FE model, we reconstructed an intact femur FE model and performed an analysis to compare our model with published experimental data [15]. A vertical load of 1500 N was applied on the femoral head. The axial stiffness based on our FE computation was 0.54 kN/mm and was in the measurement interval (0.76 ± 0.26 kN/mm) [15]. The individual differences showed that the FE model was satisfactorily validated.

### Boundary and loading conditions

The boundary condition was set as a fixation at the distal end of the femur, and the displacement along the *x*-, *y*- and *z*-axes at that site was set to zero [16]. Our study simulated the forces acting on the hip during the stance phase of walking [17]. A 2000 N vertical load was applied to the femoral head [18, 19]. The contact between the two fracture surfaces was defined according to the contact method described in the references, and the coefficient of friction was 0.46 [20]. The friction coefficient was 0.3 for bone-implant interactions [20] and 0.2 for implant-implant interactions [21].

**Fig. 3:**
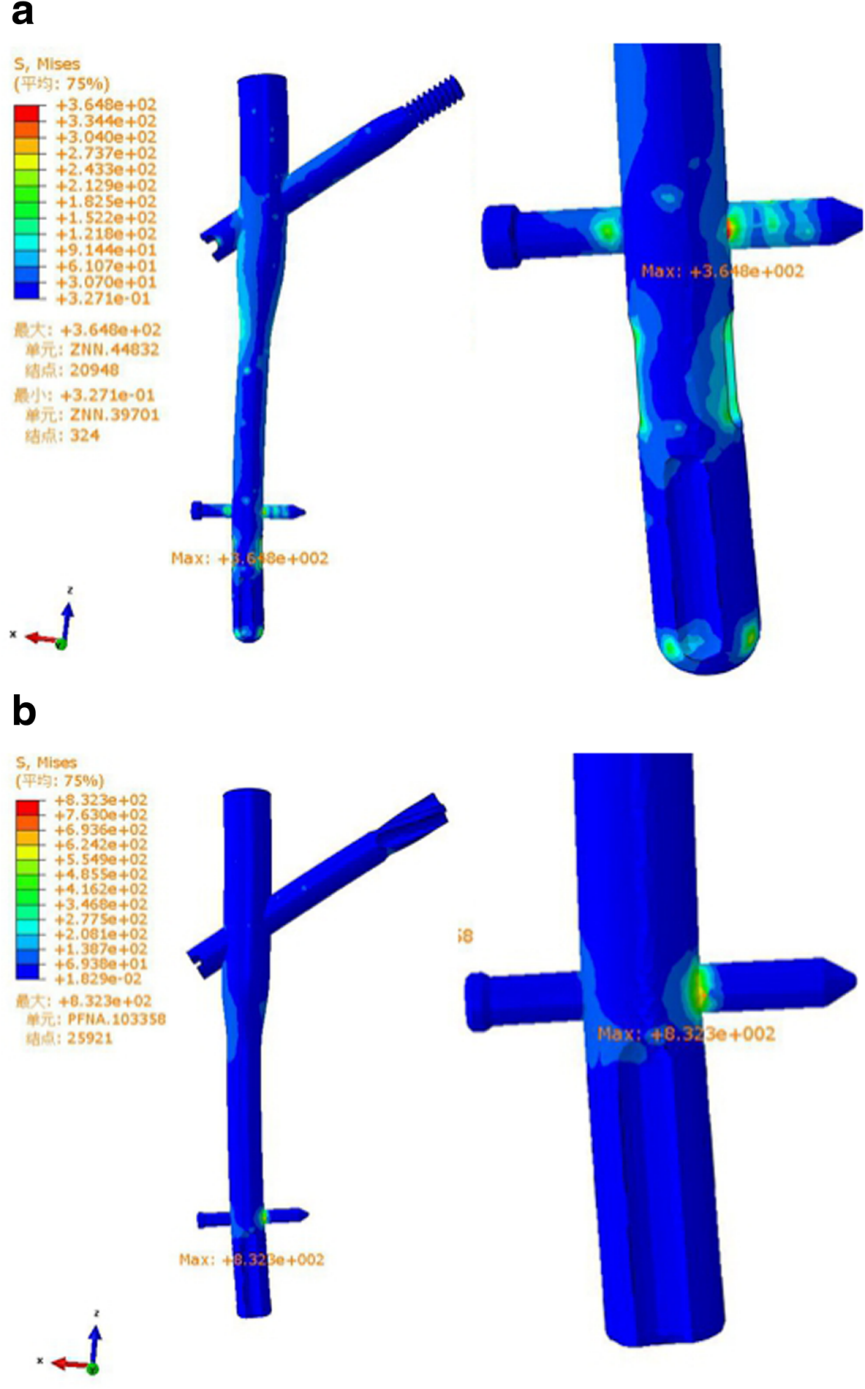
Stress distribution, peak and position analysis for internal fixation (**a** ZNN model, **b** PFNA-II model)

### Observation index

First, the von Mises stress distribution, peak value and position of the implants and femurs were obtained. Second, the displacement distribution, maximum amount and position of the models and implants were analysed. Third, the maximum displacement of the fracture end of the fracture was measured.

## Results

### Stress distribution

Differences in stress distribution were observed on the two implants and the femur. In the two implants, the stress was concentrated at the distal locking screw of each group, and the peak von Mises stresses were 364.8 MPa and 832.3 MPa in the ZNN and PFNA-II, respectively (Table 3 and Fig. 3).

**Table 3:** Parameters results

Parameters	ZNN	PFNA-II
The maximum von Mises peak stress of the implant (MPa)	364.8	832.3
The maximum von Mises peak stress of the femur (MPa)	171.8	1795.0
The maximum displacement of the model (mm)	3.768	3.713
The maximum crack distances of the fracture surface (mm)	0.08	0.07

**Fig. 4:**
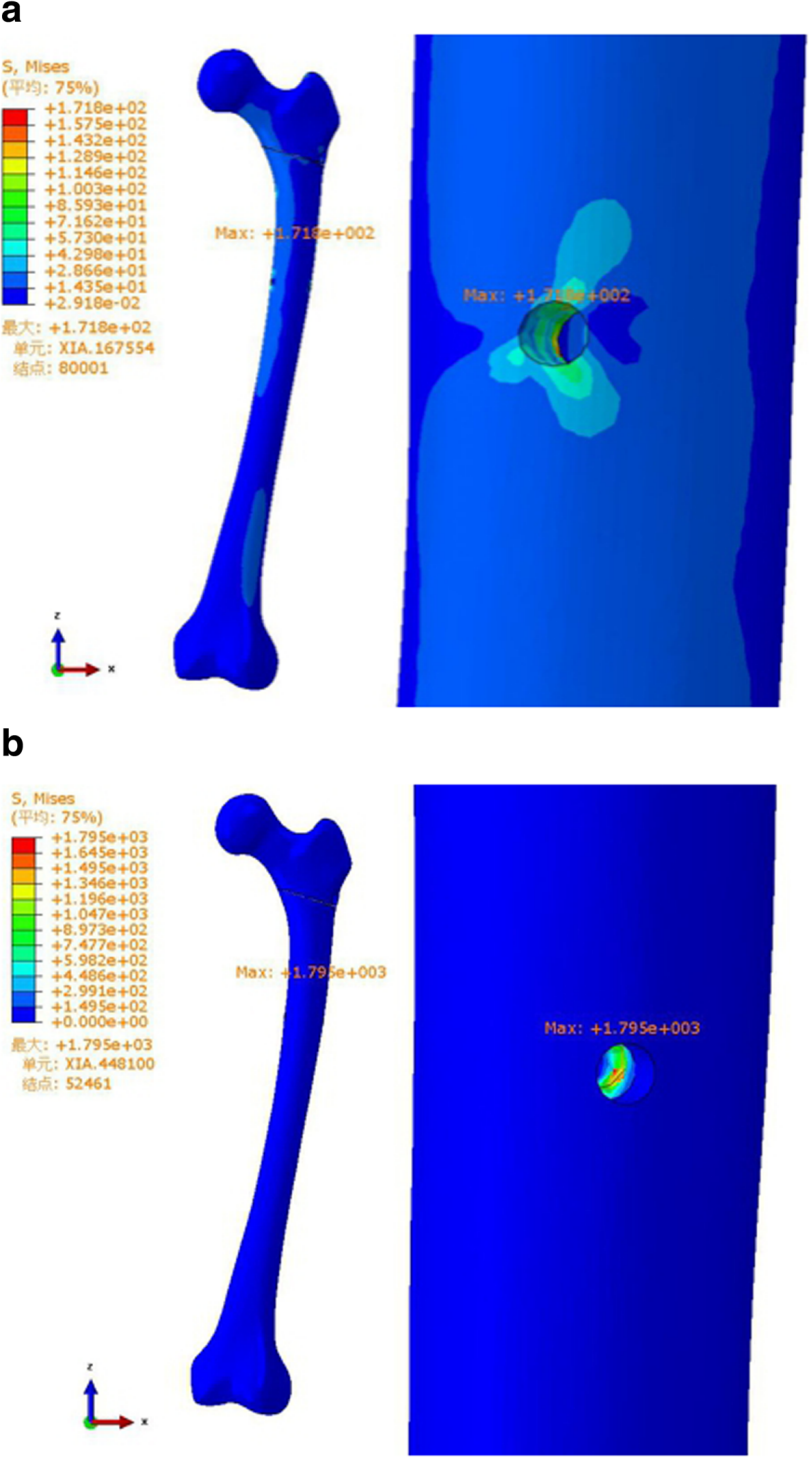
Stress distribution, peak and position analysis for femur (**a** ZNN model, **b** PFNA-II model)

In the femur, the stress was concentrated at the distal transfixation screw hole of each group, and the peak von Mises stresses were 171.8 MPa and 1795.0 MPa in the ZNN and PFNA-II, respectively (Table 3 and Fig. 4).

### Displacement

**Fig. 5:**
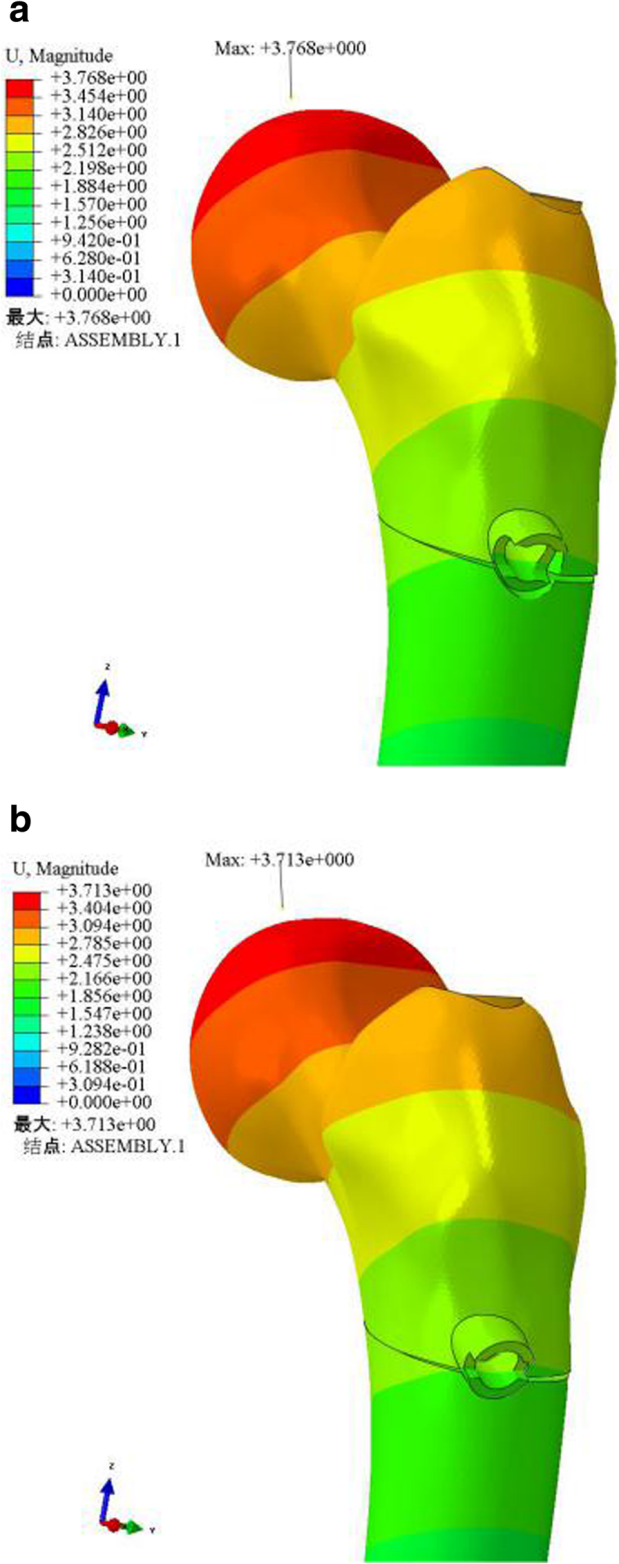
Displacement distribution, maximum amount and position of two models (**a** ZNN model, **b** PFNA-II model)

According to the displacement distribution cloud charts of the model, the maximum amount of displacement of the two models (A: ZNN model, B: PFNA-II model) was 3.768 mm and 3.713 mm, respectively, and both displacements were located at the top of the femoral head. The maximum crack distances of the fracture surfaces of models A and B were 0.08 mm and 0.07 mm, respectively (Table 3 and Fig. 5).

## Discussion

According to the data released by the National Bureau of Statistics of China, by the end of 2018, the population in China at or above the age of 60 was 249.49 million, accounting for 17.9% of the total population. Among them, 166.58 million were aged 65 or above, accounting for 11.9% of the total population. These data show that China has become an ageing society. With the ageing of societies, the incidence of hip fracture in the elderly is increasing year by year, which has become a serious social problem that threatens the health and even the life of the elderly population all over the world [22, 23]. How to improve the treatment effect and long-term functional recovery after hip fracture in elderly patients has become one of the popular topics and challenges in the field of orthopaedics.

In reverse obliquity intertrochanter fracture, the fracture line either passes through the femoral calcar above the lesser trochanter or directly through the lesser trochanter, which destroys the integrity of the medial femoral arch and loses mechanical support. Because it is an unstable intertrochanteric fracture, reverse obliquity intertrochanter fracture can easily cause coxa vara under an axial load. In recent years, with the promotion and application of minimally invasive techniques, new instruments and the concept of enhanced recovery after surgery (ERAS) [24, 25], an increasing number of intertrochanteric fractures in elderly patients have been treated with intramedullary devices [26]. The practice guidelines from the American Association of Orthopaedic Surgery (AAOS) [27] support the use of these devices, especially for unstable fractures.

Research on the treatment of intertrochanteric fractures with intramedullary nails has been focused mainly on PFNA/PFNA-II (Depuy Synthes, USA), Gamma3 (Stryker, USA) and InterTan (Smith & Nephew, UK), but few on the ZNN. Shin et al. [8] compared the ZNN and PFNA-II by conducting prospective randomised controlled studies and found that the operative time and intraoperative fluoroscopy time of the ZNN group were longer than that of the PFNA-II group, with statistically significant differences (*P* < 0.05), while there were no statistically significant differences in the hip function score, screw cutting rate, reoperation rate, TAD and other aspects (*P* > 0.05). The major difference in the design feature between the two nails was the geometry of the cephalocervical screw (lag screw versus helical blade) and main nail. For the ZNN, both the short and long nails had an anterior bow design, while the short nails of PFNA/PFNA-II, gamma 3 and InterTan were straight nails without an anterior bow design.

However, as far as we know, no biomechanical studies have compared the ZNN with PFNA-II in the treatment of reverse obliquity intertrochanter fracture. FEA is a commonly used mechanical evaluation method in orthopaedics. In this study, we constructed 3D finite element models of the ZNN and PFNA fixation methods for the treatment of reverse obliquity intertrochanter fractures in elderly patients to compare the differences in biomechanical properties of bone treated using these methods. In addition, we placed a cephalomedullary screw in the centre of the femoral neck and the femoral head in both groups and controlled the TAD to be within 20 mm (17.52 mm in the ZNN group and 19.33 mm in the PFNA-II group) to reduce deviations that may affect our research results.

In this study, we found that under the same load, both models produce different stress distributions, but the maximum displacement was similar. The stress of the implant and femur in the PFNA-II model were higher than that in the ZNN model, and the implant stress was concentrated at the distal locking screw of each group. This finding may explain the breakage of the distal locking screw and the femoral shaft fracture of PFNA-II observed in the study of Sawaguchi et al. [7]. The stresses in the implant and bone were reduced with the ZNN. The explanation for this reduction may be that the ZNN is designed to anatomically fit to the femur, providing better support to the fracture fragments and better distributing stress transduced by the bone along the intramedullary nail. These qualities may reduce the risk of implant and/or bone breakage.

There were still some shortcomings in our current study. First, the actual stress on the femur is complex. The simplified model adopted in this experiment could not fully reflect the actual stress. Second, there are many types of AO classification of intertrochanter fracture of the femur, and only one subtype, 31-A3.1, was simulated in this study. Finally, the femur and implants are anisotropic materials. However, in this study, they were simplified into homogenous, isotropic and elastic materials.

## Conclusion

Through FEA, we found that the displacement in the two models (ZNN and PFNA-II) was similar, but the stresses in the implant and bone were reduced with the ZNN. Therefore, the ZNN implant may provide biomechanical advantages over the PFNA-II implant in reverse obliquity intertrochanteric fractures. These findings from our study may provide a reference for the perioperative selection of internal fixations. Because FEA is a simulation analysis, our results also need to be confirmed by in vitro biomechanical experiments and prospective multi-centre clinical randomised controlled trials.

## Data Availability

Please contact author for data requests.

## Abbreviations

FEA: Finite element analysis
ZNN: Zimmer natural nail
PFNA-II: Proximal femoral nail antirotation-II
PFNA: Proximal femoral nail antirotation
3D: Three dimensional
CAD: Computer-aided design
FE: Finite element
TAD: Tip-apex distance
ERAS: Enhanced recovery after surgery
AAOS: American Academy of Orthopaedic Surgeons
