# Correction to: Finite element analysis of two cephalomedullary nails in treatment of elderly reverse obliquity intertrochanteric fractures: zimmer natural nail and proximal femoral nail antirotation-ΙΙ

**DOI:** 10.1186/s13018-020-01612-x

**Published:** 2020-03-09

**Authors:** Jian Chen, Jian-xiong Ma, Ying Wang, Hao-hao Bai, Lei Sun, Yan Wang, Bin Lu, Ben-chao Dong, Ai-xian Tian, Xin-long Ma

**Affiliations:** 1 Department of Orthopedics, Tianjin Medical University General Hospital, Tianjin, 300052 People’s Republic of China; 2 Institute of Orthopedics, Tianjin Hospital, Tianjin University, Tianjin, 300050 People’s Republic of China

**Correction to: J Orthop Surg Res**

**https://doi.org/10.1186/s13018-019-1468-3**

In the original publication of this article [1], Chinese text of the Figs. 3, 4, 5 has not been converted to English.

Corrected figures are shown below.

**Fig. 3:**
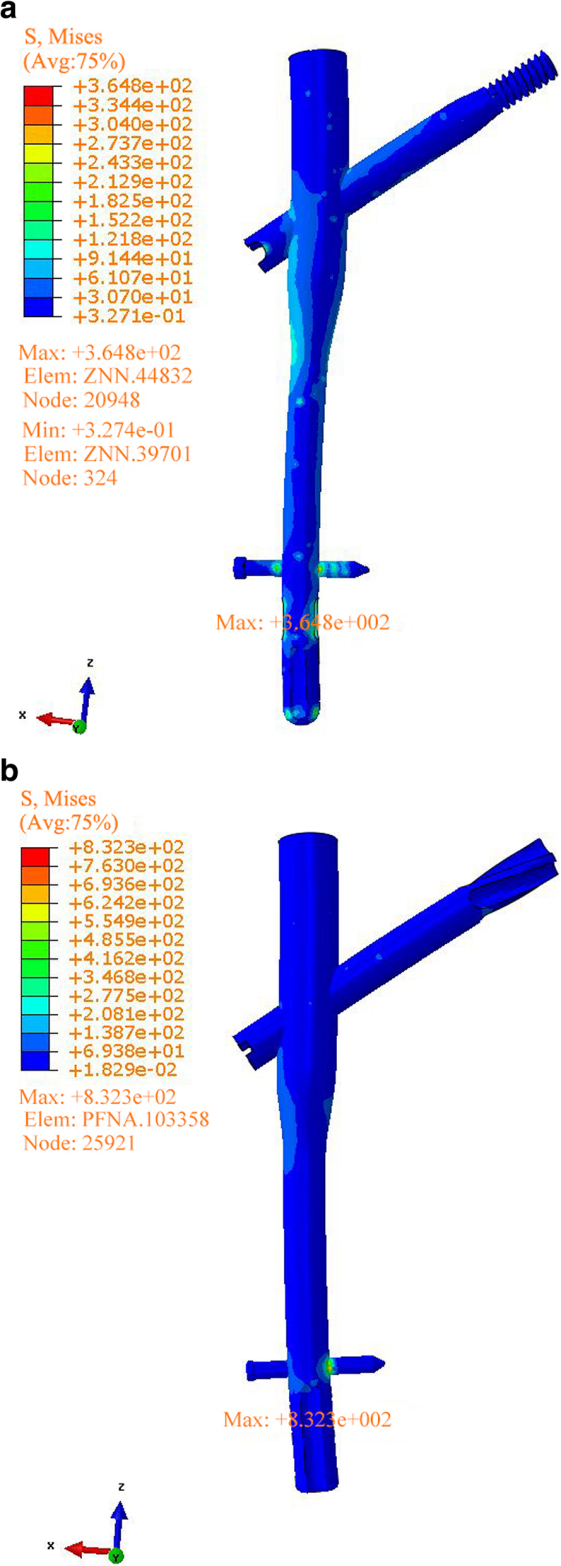
Stress distribution, peak and position analysis for internal fixation (**a** ZNN model, **b** PFNA-II model)

**Fig. 4:**
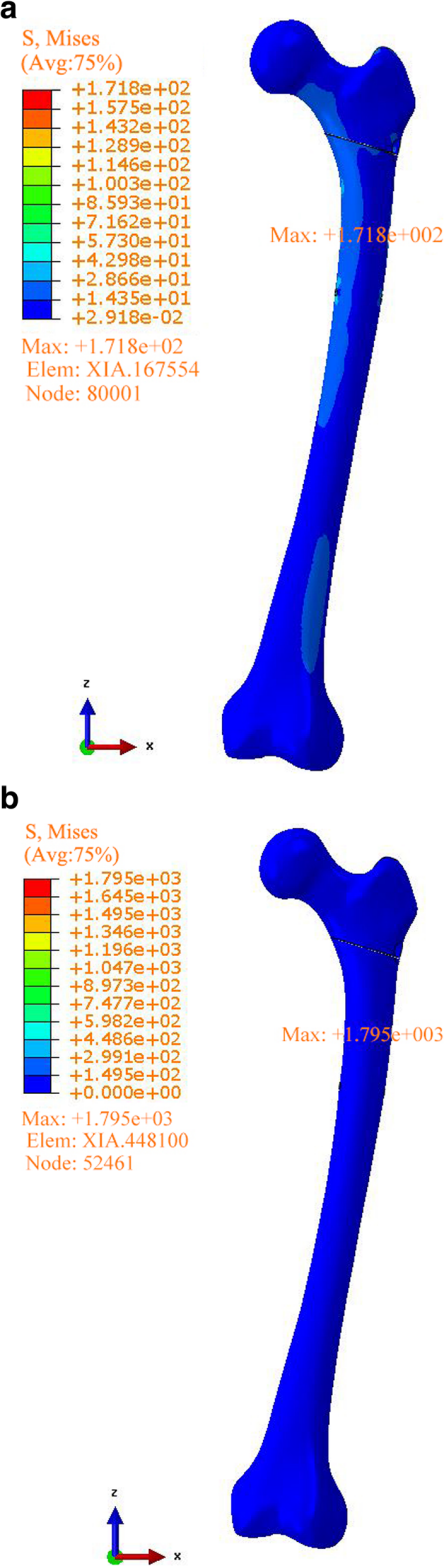
Stress distribution, peak and position analysis for femur (**a** ZNN model, **b** PFNA-II model)

**Fig. 5:**
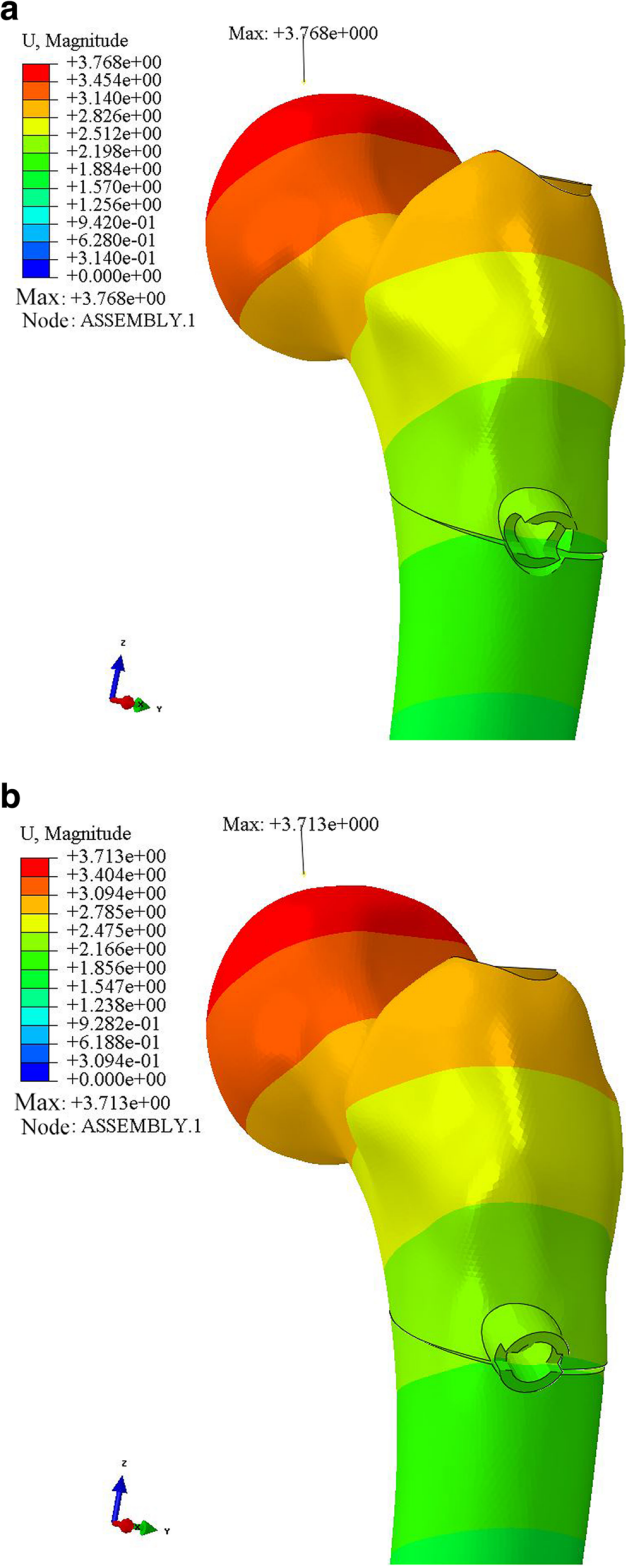
Displacement distribution, maximum amount and position of two models (**a** ZNN model, **b** PFNA-II model)
